# The impact of childhood trauma on emotional distress and the moderating role of sense of coherence among college students in China

**DOI:** 10.1038/s41598-024-60537-1

**Published:** 2024-04-29

**Authors:** Ningdan Fan, Huanhuan Fan, Ruiqing Luo, Yu Wang, Yushun Yan, Xiao Yang, Min Wang, Yikai Dou, Rongjun Ni, Jinxue Wei, Wanqiu Yang, Xiaohong Ma

**Affiliations:** 1 Mental Health Center and Laboratory of Psychiatry, West China Hospital of Sichuan University, Chengdu, 610041 China; 2 Chongqing Mental Health Center, Chongqing, China; 3 School of Ethnology and Sociology, Yunnan University, Kunming, China; 4 School of Medicine, Yunnan University, Kunming, China

**Keywords:** Childhood trauma, Sense of coherence, Moderating effect, Mental health, College students, Post-traumatic stress disorder, Human behaviour

## Abstract

Childhood trauma is strongly linked to emotional distress. However, few studies have explored the impact of sense of coherence (SOC) on the relationship between childhood trauma and emotional distress in college students. This study aimed to explore its impact on the relationship between childhood trauma and emotional distress. Analyzing data from 2307 Chinese college students, we found that SOC moderated the association between childhood trauma and anxiety/depression levels. Females showed higher SOC and lower anxiety/depression despite experiencing more childhood trauma. Multiple linear regression revealed that anxiety was negatively associated with SOC(*P* < 0.001) and grade(*P* = 0.027), and positively with childhood trauma(*P* < 0.001) and male gender(*P* = 0.004). Similarly, the depression exhibited similar associations. SOC moderated negatively the relationship between CTQ and anxiety, as well as between CTQ and depression. Childhood trauma is associated with increased emotional distress risk among college students, but a strong SOC can reduce this risk.

## Introduction

Leaving home to attend post-secondary school can pose significant challenges, including stress, depression, and anxiety, particularly for freshman, as they may struggle to adjust to their new environment^1^. Various external factors can contribute to emotional problems among college students, including financial hardships, social isolation, relationship challenges, discrimination and physical health problems^2,3^. The pressure to obtain a college degree, often viewed as a key marker to success, further complicates these challenges^1^. The internal factors may arise from childhood trauma, which could manifest as a sense of displacement upon leaving home for college^4^.

It is during this developmental transition that the consequences of childhood emotional maltreatment may become salient for college students^5^. College students who have experienced child maltreatment may have developed maladaptive coping strategies in an effort to cope with the effects of their past abuse^5,6^. For example, college students who experience emotional neglect in childhood may feel that they are poor and unworthy of being loved and have difficulty trusting others. Therefore, they are unable to participate well in interpersonal interactions, which means that they engage in fewer prosocial behaviors in college.

The concept of sense of coherence (SOC) encapsulates an individual’s cognitive predisposition towards life, encompassing the perception of life events as identifiable, foreseeable, and explicable. It entails a belief in one’s capacity to navigate stress through internal and external resources, viewing challenges as avenues for growth and achievement^7^. Collectively, these facets form the holistic SOC framework, shaping individuals’ cognitive evaluations of life occurrences and guiding their coping mechanisms and psychological responses in the face of adversity^8^. Previous studies have found that individuals with higher SOC levels tend to report fewer negative life events and psychological symptoms. Furthermore, while a greater number of negative life events correlated with a higher incidence of physical disorders in general, this association was particularly evident among individuals with a weaker SOC^9^.

Resilience refers to an individual’s ability to recover and adapt in the face of adversity, stress, or trauma. There is a close relationship between SOC and resilience^10^. SOC is a resource that enhances resilience to use adaptive coping strategies to buffer the negative effects of stress, developing positive subjective well-being^8^. Specifically, individuals with a higher SOC tend to better cope with life challenges because they tend to view life events as comprehensible, manageable, and meaningful. This positive cognitive orientation helps them maintain psychological stability, thereby mobilizing resources, seeking support, and ultimately overcoming difficulties more effectively. Therefore, SOC can be seen as an important internal factor in the development of resilience, promoting individuals’ adaptation and growth in the face of adversity^8^. Given the prevalence of childhood trauma and emotional problems in college students, it is imperative that we continue to examine the role of SOC as a promotive and protective factor.

Childhood maltreatment is a significant concern in China. Previous studies have found that 36.6% of the population reported experiencing physical child abuse^11^. Additionally, a study specifically focused on the prevalence of childhood sexual abuse of college students was reported to be 24.8% in female and 17.6% in male participants, respectively^12^. The most common types of childhood trauma reported in Chinese college students were emotional neglect, physical neglect, and psychological abuse^13–16^. Furthermore, childhood maltreatment has also been identified as a risk factor of relevant psychological outcomes in college students. Previous studies have consistently found that the experience of physical and sexual abuse in childhood would increase the risk of various psychological and behavioral disorders, such as depression, anxiety, substance abuse and personality disorders^17–21^. Subsequent research has found similar consequences for emotional abuse (psychological abuse but no physical harm) and emotional neglect (emotional deprivation or lack of emotional support), which confer psychological and behavioral problems such as depression. It is important that effective strategies are implemented to prevent and address childhood trauma, as well as to provide support and resources for those who have experienced it.

However, to our best knowledge, existing literature did not thoroughly investigate this hypothesis among college students through the lens of SOC, especially in China during COVID-19 restrictions. Therefore, this study aims to examine the effects of childhood traumatic experiences on negative emotions including depression and anxiety, and furthermore, to explore the role of SOC in this process among Chinese college students.

## Materials and methods

### Participants

This cross-sectional study was conducted among college students in southwest China from December 2020 to January 2021. The survey was created and distributed using an online survey website (http://www.wjx.cn). Inclusion criteria were as follows: undergraduates in China took part in the study voluntarily and native Chinese speakers. The exclusion criteria were as follows: the questionnaire information is incomplete or the occupation is not a student. A total of 2334 participants were enrolled in this study. Among them, 19 questionnaires were excluded due to occupation not being categorized as “student,” and 8 were disqualified due to missing general information pertinent to the study content, including gender and age. Consequently, 2307 questionnaires were deemed valid, resulting in an effective rate of approximately 99%.

All participants were provided with a comprehensive study description and provided informed consent online. The study was approved by the corresponding Institutional Review Board (IRB) of the Institute of Psychology (A20021), Chinese Academy of Sciences, and the whole process was performed in conformity with the Declaration of Helsinki.

### Assessments

#### Sense of coherence (SOC)

The sense of coherence (SOC) scale used in this study is an abbreviated version of the SOC-13 self-report questionnaire, which measures the level of SOC^7,22^. This questionnaire is a 7-point Likert scale which is semantic and differential scale with a positive attribute at one endpoint and a negative attribute at the other endpoint. The SOC total score ranges from 13 to 91, with higher scores indicating a stronger sense of coherence. We used the total score for analysis in our main text. The Chinese version of the SOC scale has satisfactory reliability as well as convergent and discriminant validity^23–25^. The internal consistency for SOC was pretty good in this sample (ω = 0.89, Cronbach’s α = 0.794)^26,27^.

#### Childhood trauma questionnaire (CTQ)

The CTQ is a 28-item self-report questionnaire which was used to evaluate traumatic experiences in childhood (before 16 years old), including various forms of abuse and neglect^28,29^. The CTQ comprises 25 clinical items, where scores are counting towards the total, and 3 validity items. It is a 5-point Likert scale ranging from 1 (“never”) to 4 (“always”). We used the total score of CTQ for analysis in our main text, five subscales were also analyzed in our Supplementary materials. The CTQ has good reliability and validity in Chinese college student samples, and exhibited high internal consistency in this study (ω = 0.96, Cronbach’s α = 0.845)^27,30,31^.

#### Generalized anxiety disorder questionnaire (GAD-7)

The GAD-7 scale is a 7-item version used to screen for generalized anxiety disorder (GAD) and assess its severity in respondents over the past two weeks^32^. The scale is a 4-point Likert scale (ranging from 0- “not at all” to 3- “nearly every day”). The cutoff point score of the scale is 5^33^. In this study, internal consistency of GAD-7 was excellent ^34^(ω = 0.94, Cronbach’s α = 0.930)^27^.

#### Patient health questionnaire 9 (PHQ-9)

The PHQ scale is a 9-item version, which is widely used to measure for assessing depression severity over the past two weeks with good reliability and validity. The scale is a 4-point Likert scale (ranging from 0- “not at all” to 3- “nearly every day”). The cutoff point score of the scale is 5^35^. In our sample, internal consistency of PHQ-9 was excellent (ω = 0.93, Cronbach’s α = 0.914)^27^.

### Statistical analysis

Statistical analyses were performed by the Statistical Package for the Social Sciences (SPSS) 24.0 and R software (version 4.6), and a significance level of 0.05 was set for all two-tailed tests. T-tests were used to assess the statistical differences of sample characteristics between male and female. To explore the overall relationship among multiple variables, we applied path analysis to test the relationship among interrelated study variables in a hypothesized model. In our model, GAD-7 and PHQ-9 were modeled as outcome variables, while CTQ was modeled as an observed variable, and SOC was modeled as a moderator. Path analysis estimated both the direct and indirect effects one variable had on the outcome variable. Third, we conducted an analysis of the moderating effect^36^, which takes CTQ as the independent variable, GAD-7 or PHQ-9 as the dependent variable, and SOC as the moderating variable among them, so as to further explore the impact of SOC on the relationship between CTQ and GAD-7/PHQ-9. Multiple regression analysis was performed to examine the relationship between mental health problems, SOC, and other causes. A two-sided p < 0.05 is regarded as statistically significant, with * indicating p < 0.05, whereas **p < 0.01, ***p < 0.001 in the current study.

## Results

### Sociodemographic characteristics

A total of 2307 college students were included in the study, comprising 1012 freshman and 1295 upperclassmen (723 sophomores, 555 juniors, and 17 seniors) from 16 colleges in Southwest China and eastern coastal areas. Of the participants, 1263 (54.7%) were male, and 1044 (45.3%) were female. The mean (SD) age of participants was 19 (1.29) years (range: 17–26), with no significant gender difference (Table 1).

**Table 1:** Sociodemographics and clinical characteristics by gender.

Item	Total (n = 2307)	Male (n = 1263)	Female (n = 1044)	T	*P*
	Mean (SD)	Mean (SD)	Mean (SD)		
Age (years)	19.61 (1.29)	19.64 (1.31)	19.57 (1.26)	1.149	0.251
SOC	57.36 (9.85)	56.46 (9.38)	58.45 (10.30)	− 4.809	< 0.001
GAD-7	3.29 (3.94)	3.58 (3.88)	2.93 (3.98)	3.986	< 0.001
PHQ-9	4.85 (4.75)	5.16 (4.60)	4.48 (4.90)	3.392	< 0.001
CTQ	37.13 (12.65)	36.21 (11.71)	38.24 (13.62)	− 4.212	< 0.001
Emotional abuse	6.83 (2.98)	6.84 (2.90)	6.82 (3.08)	− 0.719	0.472
Physical abuse	5.91 (2.57)	5.68 (2.34)	6.17 (2.81)	− 4.835	< 0.001
Sexual abuse	5.70 (2.44)	5.59 (2.22)	5.83 (2.67)	− 2.390	0.017
Emotional neglect	10.50 (5.72)	10.15 (5.38)	10.91 (6.09)	− 2.778	0.006
Physical neglect	8.19 (3.30)	7.94 (3.14)	8.50 (3.45)	− 4.059	< 0.001

Data are presented as mean (SD).

*SOC* sense of coherence, *GAD-7* Generalized Anxiety Disorder Questionnaire 7, *PHQ-9* Patient Health Questionnaire 9, *CTQ* Childhood Trauma Questionnaire. T-tests were conducted to compare the gender differences in clinical characteristics.

### Gender differences in clinical characteristics

Females have higher levels of SOC than males, particularly in comprehensibility and manageability, while no significant gender difference is observed in meaningfulness. Moreover, females have more significant childhood trauma than males, including physical abuse, sexual abuse, emotional neglect and physical neglect, see Table 1.

### Multiple linear regression models

**Figure 1:**
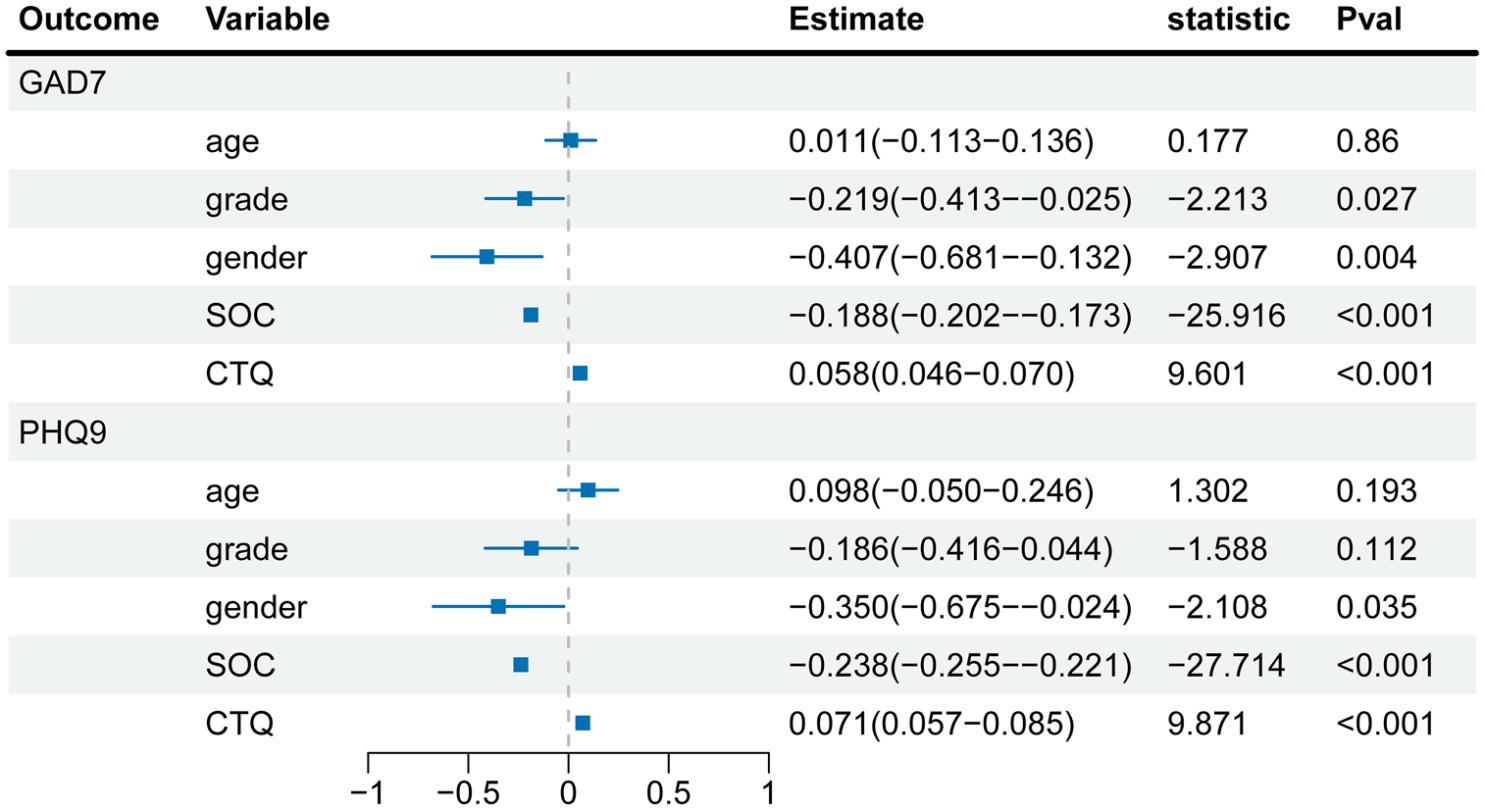
Multiple linear regression analysis of GAD-7/ PHQ-9 and five factors. GAD-7, Generalized Anxiety Disorder Questionnaire 7; *PHQ-9* Patient Health Questionnaire 9, *SOC* sense of coherence, *CTQ* childhood trauma questionnaire.

After controlling for age, gender and grade, childhood trauma was significantly associated with the severity of both anxiety (*P* < 0.001) and depression severity (*P* < 0.001). Conversely, students with low SOC were more likely to report symptoms of anxiety (*P* < 0.001) or depression (*P* < 0.001) than those with medium or high SOC. In addition, freshmen were at a higher risk of experiencing anxiety compared to upperclassmen (*P* = 0.027). Males have higher levels of anxiety (*p* = 0.004) and depression (*P* = 0.035) than females (Fig. 1).

In our path analysis, significant mediating effect was also observed for SOC in the relationship between CTQ and GAD-7/PHQ-9, please see Supplementary Table S2, however, we did not focus on this result in our discussion because we believe that the moderation effect is more important.

### The moderating effects of SOC

**Figure 2:**
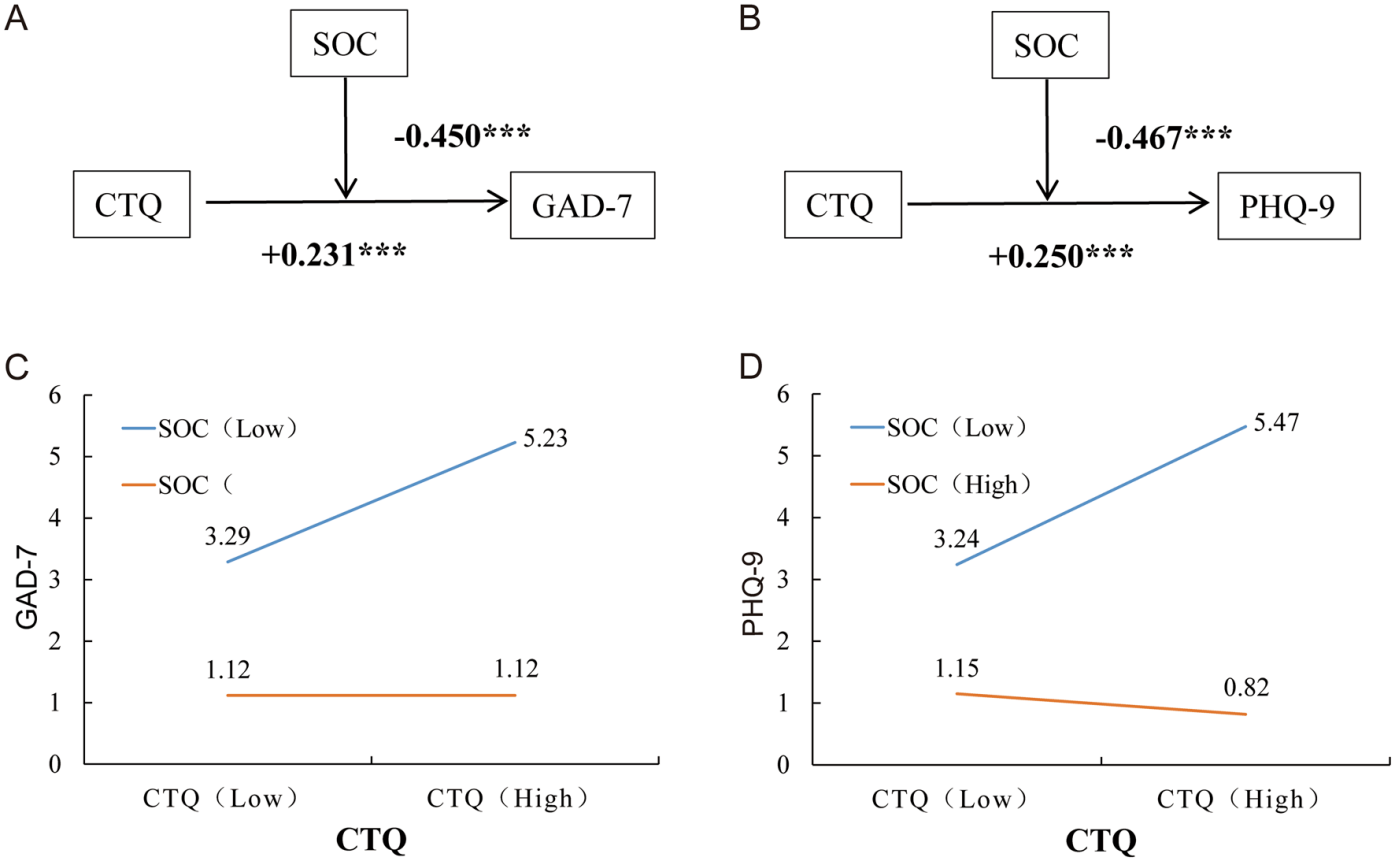
Moderating effect of SOC on the relationship between CTQ, GAD-7 (**A**, **C**) and PHQ-9 (**B**, **D**). *SOC* sense of coherence, *GAD-7* generalized anxiety disorder questionnaire 7, *PHQ-9* patient health questionnaire 9, *CTQ* childhood trauma questionnaire.

Our results showed that SOC had negative moderating effects on the association between CTQ on GAD-7 (T = − 2.905, *P* < 0.001) and PHQ-9 (T = − 3.930, *P* < 0.001) (Table 2 and Table 3). This indicates that SOC can weaken the effect of CTQ on depression and anxiety among college students (Fig. 2A,B). As shown in the moderating effect diagram (Fig. 2C,D), participants with higher scores of SOC experienced a dampened influence of CTQ on anxiety and depression, and vice versa.

**Table 2:** Moderating effect of SOC on the relationship between CTQ and GAD-7.

	Estimate	SE	T	*P*
CTQ	0.1526	0.0283	5.389	< 0.001
SOC	− 0.1248	0.0203	− 6.154	< 0.001
CTQ*SOC	− 0.0015	0.0005	− 2.905	< 0.001

*CTQ* childhood trauma questionnaire, *SOC* sense of coherence, *GAD-7* generalized anxiety disorder questionnaire 7.

**Table 3:** Moderating effect of SOC on the relationship between CTQ and PHQ-9.

	Estimate	SE	T	*P*
CTQ	0.2219	0.0333	6.665	< 0.001
SOC	− 0.1372	0.0238	− 5.755	< 0.001
CTQ*SOC	− 0.0024	0.0006	− 3.930	< 0.001

*CTQ* childhood trauma questionnaire, *SOC* sense of coherence, *PHQ-9* Patient Health Questionnaire 9.

## Discussion

A major finding in this study revealed that a childhood trauma seems to sensitize individuals. When other negative events or stress occur later in their lifetimes, they are then more prone to develop emotional distress. Our results suggest that this sensitization is partially explained by a weakening of their SOC, which is consistent with our hypothesis.

In our sample, females had more significant childhood trauma than males, which was in line with the previous studies^37,38^. Previous research has indicated that female may be more inclined to report certain types of childhood trauma, while estimates of the male sexual abuse may be influenced by under-reporting due to a reluctance to disclose such abuse^39^. Besides, consistent with the previous study, we observed that childhood trauma was one of the risk factors for depression and anxiety^29^. Researchers proposed that early adversity may lead to neural and social cognitive changes that affect the formation and enhancement of psychological congruence in subjects and may constitute a pathophysiological process underlying psychiatric disorders such as depression and bipolar disorder^40,41^. This assumption of causality is also supported by prospective studies showing that the number of people with psychiatric disorders would be reduced by one-third if childhood trauma was removed from the population as a risk factor^42^. These studies provide further support for the impact of childhood trauma on negative emotion and even psychiatric disorders such as depression and bipolar disorder.

The present study also found that female have higher SOC than male, consistent with previous study on adolescents and teachers, which also reported sex-specific differences in SOC^43,44^. Perhaps it could be attributed to females’ greater attentiveness to their thoughts and more effective self-expression. They tend to utilize available resources effectively to cope with pressure and alleviate its impact, even when experiencing similar stressors as males. This confluence of evidence underscores the need for continued exploration into potential gender disparities in the current level of SOC. This study also showed a significant negative correlation between undergraduate students’ SOC and their level of depression and anxiety. This is consistent with the results of previous studies demonstrating SOC significantly negatively predicted their anxiety and depression levels^45,46^. The relationship between SOC and negative emotions such as depression and anxiety illustrates that when individuals can show adequate and comprehensive awareness of various stresses in the outside world, are able to face and solve problems actively, and maintain confidence in life persistence, the probability that negative emotions will further progress to disease is reduced. This illustrates that the effect of cognition on negative emotions is not unilateral and involves factors such as comprehension, prediction, etc.

Our result shows that SOC exhibited a notable negative moderating effect on depression and anxiety among college students,which highlighted the pivotal role of SOC in mitigating emotional distress, aiming to offer fresh perspectives for addressing mental health concerns within this demographic. Feldt et al. proposed that a heightened level of SOC hinges upon two factors: the availability of ample resources to navigate stress and the capacity to mobilize those resources to effectively address stressors. These resources encompass physical, emotional, and cognitive elements that facilitate individual growth^47^. Childhood and adolescence mean the stage at which individual development is critical. Other researchers insisted that parenting played a pivotal role in shaping adolescents’ SOC^48–50^. Family resources and climate have a substantial influence on the perception of SOC. A good family environment and parent–child relationship are important sources of emotional resources during childhood and adolescence. Parents’ own educational attainment and ways of parenting then provide resources for children’s and adolescents’ cognitive development. In contrast, as a structural linear equation modeling about the effects of varied variables on students’ level of SOC indicated, at-risk home environments struggle to provide the developing child and adolescent with the required resources, and the emergence of cumulative risk further compromises the ability to unearth and mobilize resources during childhood and adolescence, resulting in impaired development at the level of their SOC^50^. This enlightens us that more work is needed in the future to develop children’s and adolescents’ cognitive abilities and improve parents’ educational levels and cognitive abilities. Group interventions are a well-established method for strengthening college students’ SOC. A randomized controlled trial using a CBT-based group counseling program showed significant improvement in students’ SOC scores^51^. This highlights the potential of integrating stress coping and emotion management training into the curriculum. Additionally, training programs for both students and faculty on these skills could foster a more supportive learning environment and further enhance SOC levels^52^. Research on technologies to improve SOC in college students is ongoing^53^.

While our sample was collected during the COVID-19 pandemic, this study focuses on the independent relationship between childhood trauma and emotional distress in college students during this period. In another large-scale survey of college students in China, acute stress, anxiety and depressive symptoms are prevalent during the COVID-19 pandemic. Multiple epidemic and psychosocial factors, such as family members being infected, massive media exposure, low social support, senior year and prior mental health problems were associated with increased risk of mental health problems^54^. The COVID-19 pandemic can be seen as a major stressful event, and SOC also has a protective effect on the emotions of college students during this period. It is noteworthy that certain pertinent studies have indicated that the effect of SOC on depression was modified by negative epidemic information exposure. With the increase of negative epidemic information exposure, the predictive effect of SOC on depression is increasing gradually. These findings demonstrated that negative epidemic information exposure was associated with an increased psychological distress in the sample. A high SOC also played a certain protective role in the adaptation of college students in the post-epidemic period^55^.

The current cross-sectional study has limitations in its capacity to examine the temporal relations among childhood trauma, SOC and negative emotions. Longitudinal analysis is needed for comprehensively understanding the effects of the study variables, and future studies would benefit from more extended analysis. Secondly, utilizing a more comprehensive and objective measure of SOC could provide a more nuanced insight into the relationships explored in this study. Additionally, the attempt at a full SEM analysis resulted in a poor model fit in this study. We acknowledge that relying solely on the structural model for our analysis may lead to limited interpretation of the results, as the estimates derived from this approach could be based on an imperfect measurement model. Consequently, the interpretation of our findings should be conducted with caution. Finally, due to the relatively homogenous nature of the undergraduate sample, caution should be exercised in generalizing the findings to all college students. Future studies with more diverse and representative samples are warranted, and our endeavor will involve collecting data from multiple colleges across the nation for a larger-scale survey. Our upcoming research will strive to dig deeper into exploring the contributing factors involved.

This study emphasizes that childhood trauma can increase the risk of emotion distress, and the SOC negatively moderates this process, theoretically offering a certain level of protection for college students’ mental health. University psychological education is crucial in equipping students with specific skills to enhance their SOC and mental health.

## Supplementary Information

Supplementary Tables.

## Data Availability

The datasets generated and analyzed during the present study are not publicly available due to no permission from the ethics committee, but are available from the corresponding author on reasonable request.
