# Correction: The impact of childhood trauma on emotional distress and the moderating role of sense of coherence among college students in China

**DOI:** 10.1038/s41598-025-31397-0

**Published:** 2025-12-12

**Authors:** Ningdan Fan, Huanhuan Fan, Ruiqing Luo, Yu Wang, Yushun Yan, Xiao Yang, Min Wang, Yikai Dou, Rongjun Ni, Jinxue Wei, Wanqiu Yang, Xiaohong Ma

**Affiliations:** 1 Mental Health Center and Laboratory of Psychiatry, West China Hospital of Sichuan University, Chengdu, 610041 China; 2 Chongqing Mental Health Center, Chongqing, China; 3 School of Ethnology and Sociology, Yunnan University, Kunming, China; 4 School of Medicine, Yunnan University, Kunming, China

Correction to: *Scientific Reports* 10.1038/s41598-024-60537-1, published online 29 April 2024

In the original version of this Article, Wanqiu Yang was omitted as a corresponding author. The correct corresponding authors for this Article are Wanqiu Yang and Xiaohong Ma.

The original Article has been corrected.

