# Supplementary material for: The impact of childhood trauma on emotional distress and the moderating role of sense of coherence among college students in China
Source: Sci Rep. 2024 Apr 29;14:9797. doi: 10.1038/s41598-024-60537-1 (PMC11058193; doi:10.1038/s41598-024-60537-1)
Supplement: Supplementary file 1 — Supplementary Tables. [file 41598_2024_60537_MOESM1_ESM.docx]

***Supplementary material:***

Supplementary Table S1. The omega hierarchical for each scale.

| Scale | omega |
| --- | --- |
| SOC | 0.89 |
| CTQ | 0.96 |
| GAD-7 | 0.94 |
| PHQ-9 | 0.93 |

Abbreviations: SOC, Sense of Coherence; CTQ, Childhood Trauma Questionnaire; GAD-7, Generalized Anxiety Disorder Questionnaire; PHQ-9, Patient Health Questionnaire 9.

Supplementary Table S2. Mediating effect of SOC on the relationship between childhood trauma and anxiety or depression.

| Path | Direct Effects | | | | | Indirect Effects | | |
| --- | --- | --- | --- | --- | --- | --- | --- | --- |
|  | Coefficient | SE | T | *P* | 95%CI | Coefficient | Bootstrap SE | 95%CI |
| CTQ→GAD-7 | 0.0118 | 0.0009 | 12.9221 | ＜0.0001 | 0.0100~0.0136 | 0.0067 | 0.0006 | 0.0055~0.0079 |
| CTQ→PHQ-9 | 0.0126 | 0.0009 | 14.0661 | ＜0.0001 | 0.0108~0.0143 | 0.0070 | 0.0006 | 0.0058~0.0082 |

Abbreviations: CTQ, Childhood Trauma Questionnaire; SOC, Sense of Coherence; GAD-7, Generalized Anxiety Disorder Questionnaire; PHQ-9, Patient Health Questionnaire 9.
